# Withaferin A as a Potential Therapeutic Target for the Treatment of Angiotensin II-Induced Cardiac Cachexia

**DOI:** 10.3390/cells13090783

**Published:** 2024-05-03

**Authors:** Vasa Vemuri, Nicholas Kratholm, Darini Nagarajan, Dakotah Cathey, Ahmed Abdelbaset-Ismail, Yi Tan, Alex Straughn, Lu Cai, Jiapeng Huang, Sham S. Kakar

**Affiliations:** 1 Department of Physiology, University of Louisville, Louisville, KY 40202, USA; sreevasa.vemuri@louisville.edu (V.V.); nicholas.kratholm@louisville.edu (N.K.); darini.nagarajan@louisville.edu (D.N.); 2 Department of Pharmacology and Toxicology, University of Louisville, Louisville, KY 40202, USA; dakotah.cathey@louisville.edu (D.C.); yi.tan@louisville.edu (Y.T.); lu.cai@louisville.edu (L.C.); jiapeng.huang@louisville.edu (J.H.); 3 Pediatric Research Institute, Department of Pediatrics, University of Louisville, Louisville, KY 40202, USA; ahmedabdelbasetahmedattia.ismail@louisville.edu; 4 Department of Anesthesiology and Perioperative Medicine, University of Louisville, Louisville, KY 40202, USA; 5 Brown Cancer Center, University of Louisville, Louisville, KY 40202, USA; alexstraughn@hotmail.com

**Keywords:** cardiac cachexia, cardiac disorders, cardiac dysfunction, withaferin A, angiotensin II, inflammatory cytokines, Ang II-induced cachexia

## Abstract

In our previous studies, we showed that the generation of ovarian tumors in NSG mice (immune-compromised) resulted in the induction of muscle and cardiac cachexia, and treatment with withaferin A (WFA; a steroidal lactone) attenuated both muscle and cardiac cachexia. However, our studies could not address if these restorations by WFA were mediated by its anti-tumorigenic properties that might, in turn, reduce the tumor burden or WFA’s direct, inherent anti-cachectic properties. To address this important issue, in our present study, we used a cachectic model induced by the continuous infusion of Ang II by implanting osmotic pumps in immunocompetent C57BL/6 mice. The continuous infusion of Ang II resulted in the loss of the normal functions of the left ventricle (LV) (both systolic and diastolic), including a significant reduction in fractional shortening, an increase in heart weight and LV wall thickness, and the development of cardiac hypertrophy. The infusion of Ang II also resulted in the development of cardiac fibrosis, and significant increases in the expression levels of genes (ANP, BNP, and MHCβ) associated with cardiac hypertrophy and the chemical staining of the collagen abundance as an indication of fibrosis. In addition, Ang II caused a significant increase in expression levels of inflammatory cytokines (IL-6, IL-17, MIP-2, and IFNγ), NLRP3 inflammasomes, AT1 receptor, and a decrease in AT2 receptor. Treatment with WFA rescued the LV functions and heart hypertrophy and fibrosis. Our results demonstrated, for the first time, that, while WFA has anti-tumorigenic properties, it also ameliorates the cardiac dysfunction induced by Ang II, suggesting that it could be an anticachectic agent that induces direct effects on cardiac muscles.

## 1. Introduction

Cachexia is a multifactorial syndrome that is characterized in patients by poor appetite, weight loss, and a gradual decline in muscle strength [1]. This may develop in at least 80% of cancer patients and it is the primary cause of death between 22–30% of all cancer patients [2,3]. Cancer-induced cachexia can not only lead to skeletal muscle loss but also cause pathological changes within the heart and induce heart failure in patients [4,5]. Heart failure and other ailments may be connected in a synergistic cycle to cause morbid effects in the patients [4,5]. The release of inflammatory cytokines by tumors initiates the pathogenesis of heart failure, and the chronic inflammation further contributes to the development of heart failure [6,7,8]. The net catabolic effect due to increased protein degradation and decreased protein synthesis detrimentally affects cardiac myocytes, resulting in alterations of important functional cardiac parameters [6,7,8]. Nevertheless, the exploration of cardiac cachexia induction and a practical remedy for this condition remains insufficient. Recent findings indicate that the prevalence of cardiac cachexia ranges from 8% to 42%, depending on the disease state [9], which emphasizes the urgency to discover novel therapeutic agents mitigating cachexia’s effects.

Withaferin A (WFA) is a steroidal lactone purified from the *Withania somnifera* plant, also known as ashwagandha or winter cherry, and belongs to the family of withanolides. WFA does not only possess anti-cancer properties [10], but also anti-inflammatory and cardiac protective properties as well [11]. Previous studies have shown that WFA has a role in both preventing the invasion of ovarian cancer cells and inducing cell-cycle arrest and apoptosis [11]. Previous work from our group has demonstrated that WFA leads to autophagic cell death in ovarian cancer cell line A2780 through DNA damage [12]. Moreover, we found that mouse models xenografted with ovarian cancer cells (A2780) resulted in the onset of skeletal muscle cachexia [13]. Treatment with WFA ameliorated and restored the decline in functional muscle strength, and it was suggested that this may be due to WFA’s potential role in the canonical NF-kB pathway. In an independent study, we sought to examine ovarian-cancer-induced cardiac cachexia and the role of WFA in attenuating this condition. Not surprisingly, the metastatic ovarian cancer xenograft generated by injected ovarian cancer cells (A2780) into NSG mice prominently precipitated muscle and cardiac dysfunction, which are characteristic of cachexia and cancer-induced cardiac cachexia, respectively [14]. This was confirmed by a decline in cardiac performance, as evidenced by significant reductions in parameters such as heart weight, heart rate, fractional shortening, ejection fraction, cardiac output, and left ventricular mass. The administration of WFA to ovarian-tumor-bearing mice yielded substantive outcomes, notably marked by a restoration of the myofibrillar cross-sectional area along with a complete amelioration of cardiac dysfunction [14]. How WFA treatment can reverse cancer-induced systemic and cardiac cachexia needs to be urgently investigated, before exploring its clinical translation.

An increase in circulating levels of Ang II is known to induce both muscle and cardiac dysfunction mimicking cancer-induced cardiac cachexia [14]. In a review article on the pharmacological management of cardiac cachexia, Rolf and colleagues pointed out angiotensin receptor (ATR) blockers as the most well-studied therapeutic approaches [15]. This was supported by experimental evidence showing the preventive effect of angiotensin II receptor antagonist on cancer-induced cachexia [16,17]. It is well-known that insulin-like growth factor 1 (IGF-1) plays an important role in skeletal muscle homeostasis by maintaining skeletal muscle protein synthesis through the phosphoinositide 3-kinase (PI3K)/AKT mechanistic target of the rapamycin (mTOR) pathway and inhibiting skeletal muscle protein degradation [18]. Emerging evidence suggests that Ang II plays a major role in mediating skeletal muscle wasting through multiple pathways, including blocking IGF-1-mediated AKT/mTOR pathways and also activating NADPH oxidases (NOXs) and uncoupling nitro oxide synthase (NOS)-mediated oxidative stress, resulting in systemic inflammation [19,20,21]. Mechanistically, therefore, tumor-bearing mice displayed increased circulating angiotensin II (Ang II) levels, to induce a transformation in the MHC isoforms from adult MHCα to fetal MHCβ in cardiac myocytes, leading to a loss of cardiomyocyte contractibility [21,22,23,24,25,26].

In our studies, we established WFA’s preventive and/or therapeutic effects on cancer-induced cardiac cachexia; however, we could not definitively pinpoint whether these preventive or therapeutic effects of WFA are derived from its ability to combat tumors, thereby reducing the tumor burden, or directly from its inherent anti-cachectic properties. Therefore, in the present study, we employed the pharmacological model to directly induce cardiac cachexia with Ang II, as used by other studies [20,22,27,28], with the intent of discerning and characterizing the potential anti-cachectic properties of WFA in mice without tumors. In our studies, we show that WFA can reverse the left ventricle (LV) dysfunction (cachexia) induced by a continuous infusion of Ang II in C57BL/6 mice.

## 2. Materials and Methods

### 2.1. Ethical Approval

All the experiments involving the utilization of mice were conducted in full compliance with the guidelines established by the National Institutes of Health for the ethical treatment and handling of laboratory animals. Approval for all experimental protocols involving mice was obtained in advance from the Institutional Animal Care and Use Committee (IACUC), University of Louisville under protocol number 19653.

### 2.2. Generation of Ang II-Induced Cachectic Mouse Model

**Figure 1:**
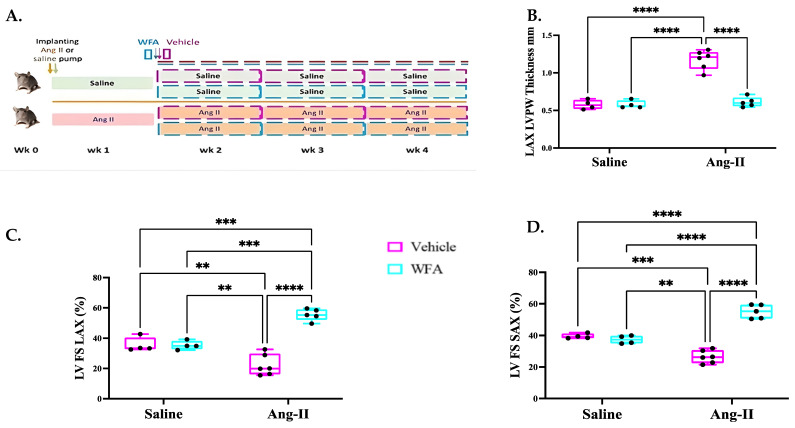
Effect of WFA on left ventricular (LV) wall thickness and systolic function in Ang II-infused mice. Illustration of experimental procedures (**A**). The graphical representation of LV posterior wall thickness in mm (**B**), LV % fractional shortening from long-axis view (**C**), and LV % fractional shortening from short-axis view (**D**). Data are presented as the mean ± SD. ** *p* < 0.01; *** *p* < 0.001; **** *p* < 0.0001 indicates a significant difference from the corresponding value of the Ang II-infused group vs. saline-infused group or Ang II–WFA group by two-way ANOVA followed by Tukey’s multiple comparison test. LV = left ventricle; LAX = long axis; SAX = short axis; FS = fractional shortening; PW = posterior wall. Four animals randomly were selected from saline infused groups and 5 to 6 animals randomly were selected from Ang II infused groups for Echo analysis.

To discern whether WFA effects on cardiac muscles were dependent upon its anti-tumorigenic properties or directly acting on cardiac muscle, a pharmacological model of cachexia independent of a tumor was employed by continuous infusion of Ang II [24,29]. Eleven-week-old female C57BL/6J mice were obtained from Jackson Laboratory (Bar Harbor, ME, USA 04609). Mice were randomly stratified into two groups, and then baseline recordings were obtained for body weight, forelimb strength, and total grip strength. Animals were implanted with osmotic minipumps (Alzet model 1004) (Cupertino, CA, USA 95014) in the suprascapular region [28]. The minipumps were loaded with Ang II (Sigma-Aldrich, St. Louis, MO, USA 68178) to deliver 1000 ng/kg/min or an equivalent volume of sterile saline and adjusted to deliver at a rate of 0.1 μL per hour for 4 weeks. After one week of implantation of pumps, the mice in both groups were further stratified to receive i.p. injections of WFA (4 mg/kg) or vehicle (10% DMSO, 90% Glycerol Trioctanoate) (Sigma-Aldrich, St. Louis, MO, USA 68178) once every three days for the last 3 weeks of total 4 weeks of study, as described previously [13,14] (Figure 1). The experimental protocol for the analysis of heart functions was repeated, and data were pooled.

### 2.3. Echocardiography and Functional Analysis of Left Ventricle

Transthoracic echocardiography was performed to assess the LV systolic and diastolic functions using a high-resolution Vevo 2100 system, equipped with a 40 MHz linear probe transducer. During the procedure, mice were placed in a supine position on a heated imaging platform to maintain body temperature at 37 °C. Recording of the heart and breathing rate as well as an echocardiogram (ECG) were performed as described previously [30]. Before the echocardiographic examination, mice were anesthetized using isoflurane administered via the use of an initial induction chamber at 3% with 1.5–2.5 L/min O_2_ flow, followed by a nose cone with 1–2% with 1.5–2.5 L/min O_2_ flow. The depth of anesthesia was monitored by assessing the absence of a toe-pinch reflex. Hairs on the skin of the thorax were carefully shaved, followed by hair removal cream to ensure complete absence of hair from the transducer site. The echocardiography transducer was applied to the chest area of the mouse, and a coupling gel was used to ensure good acoustic contact without exerting excessive pressure. Two-dimensional (2D) imaging was performed to obtain a parasternal long-axis view, parasternal short-axis view, aortic valve short-axis view, and apical four-chamber view to visualize LV structures, such as LV internal diameter, LV posterior wall thickness, and functions (LV fractional shortening, LV mitral annulus systolic velocity S’, and LV mitral annulus E/A ratio).

### 2.4. Histological Analysis

Heart samples from each group of animals were transversely sectioned before being mounted in OCT, and then stored at −80 °C until use. Sections were cut to a thickness of 5 microns using Epredia Microm HM525 cryostat at −20 °C and mounted on glass microscope slides (two sections on each slide). Slides were subjected to hematoxylin and eosin staining (H&E) for structural determination, and Masson’s trichrome staining for collagen deposition as an index of fibrosis. Staining was performed with the help of the University of Louisville Hospital Pathology Lab. Imaging of each section was performed using the 3D HISTECH Panoramic Slide Scanner following the manufacturer’s protocol for slide preparation and imaging. For image analysis and quantification of fibrosis, the MATLAB software was utilized due to its ease in segmenting colors and analyzing stains efficiently (MATLAB Version: 9.13.0 (R2022b; Update 2)). Wheat germ agglutinin (WGA) labeling was used to determine cross-sectional areas of myocyte cells. FITC conjugate (Sigma-Aldrich) was diluted in PBS to a final concentration of 50 µg/mL. Sections were incubated for 45 min, protected from light. After three washes with PBS, the sections were cover-slipped with a water-soluble antifading mounting medium. WGA imaging was performed using a Nikon Eclipse Ti confocal microscope. Images were captured and transferred to Image J (version 1.53) analysis software for quantification of cardiomyocyte cell area. Five to seven fields from each section and 15 cells from each field were analyzed.

To analyze the levels of expression of NLRP3, ASC, and Caspase 1 proteins, immunofluorescence technique was performed. Briefly, frozen OCT heart sections were fixed by incubating the sections in cold acetone (−20 °C) for 10 min followed by air-drying for 10 min at room temperature. The sections were hydrated using PBS/0.01% Tween-20 and permeabilized by incubating with 0.3% Tween-20 in PBS for 10 min. Sections were blocked with 5% bovine serum albumin for 1 h at room temperature, followed by incubation with specific antibody at 4 °C for overnight. Antibody used were for NLRP3 (cat MAB7578-SB from R&D Systems), ASC (cat # NBP1-78978 from Novus), and Casp1 (Cat # ab74279), respectively, with appropriate dilution according to instructions from the suppliers. Sections were rinsed three times with PBS (5 min each) and incubated with labeled secondary antibody (Alexa Fluor 488/568 labeled goat anti-mouse IgG and/or goat anti rabbit-IgG (1:500) (Thermo Fisher Scientific, Life Technologies Corp., Grand Island, NY, USA) for 45–60 min. The sections were air-dried and mounted in mounting medium (Eukitt Quick hardening; Sigma Aldrich, St. Louis, MO, USA). Approximately 5 representative images covering the complete section were captured (at 40× magnification) using Nikon (Eclipse TI) laser scanning confocal microscope and NIS Elements AR software (version 4.5.1). Background noise interference during confocal microscopy was maintained at minimum threshold limits by setting imaging parameters precisely and maintaining uniform parameters throughout imaging.

For identification of infiltration of macrophages in cardiac tissues, heart’s frozen sections were fixed in 4% PFA (paraformaldehyde) at room temperature and treated with hydrogen peroxide to remove endogenous peroxidase. After blocking with bovine serum albumin, the sections were incubated with anti-F4/80 rabbit monoclonal antibody (1:200, 70076, Cell Signaling Technologies, Beverly, MA, USA) at 4 °C overnight; then, horseradish peroxidase (HRP)-coupled goat anti-rabbit secondary antibody (1:500, 7074, Cell Signaling Technologies) was applied for 2 h at room temperature. Color development was carried out using 3,3′-diaminobenzidine (DAB), and nuclei were counter-stained using hematoxylin. The sections were then dehydrated, cleared, and mounted. The sections were examined under the Olympus microscope and photographed.

### 2.5. Total RNA Extraction and Quantitative Real Time PCR (qRT-PCR)

Total RNA from cardiac tissues was extracted and purified using the RNAeasy Fibrous Tissue Mini Kit from Qiagen (cat # 74704) (Germantown, MD, USA 20874) according to the manufacturer’s instructions. Briefly, approximately 25–30 mg of heart tissue was homogenized using a polytron homogenizer using the tissue extraction buffer from the kit. The total RNA was purified using the column provided in the kit following the manufacturer’s instructions. The total RNA was quantitated using a nanodrop UV–Vis spectrophotometer (Fisher Scientific). The first-strand cDNA was synthesized using a commercially available cDNA synthesis kit (iScript cDNA Synthesis Kit from BioRad Cat # 170-8891) (Hercules, CA, USA 94547) using 1 µg of total RNA for each sample. Quantitation of mRNA expression was analyzed using the SYBR Green dye and specific primers (Table 1) for each gene in the CFX-Connect Real-Time System (Bio-Rad, Hercules, CA, USA 94547). Beta-actin gene primers were used as an internal control to normalize the levels of expression of each gene as described previously [13,14].

### 2.6. Graphical Display and Statistical Analysis

Statistical analysis of the data between groups was performed using a two-way ANOVA, followed by Turkey’s multiple comparison test post hoc analysis for comparison between 4 groups containing two experimental factors to determine statistically significant differences between groups, with GraphPad Prism 10.0.2 software for Mac OS (La Jolla, CA, USA). A *p*-value of < 0.05 was considered statistically significant, unless otherwise specified. The results were presented as box-and-whisker plots with the box comprising the first, second, and third quartiles, and samples are depicted as block dots as described previously [13,14].

## 3. Results

### 3.1. WFA Reverses LV Contractility and Systolic Function

In our current studies, we tested if increased circulating levels of Ang II are the key factors responsible for cardiac cachexia using tumor-free mice and, if so, whether the cachectic effect of Ang II could be prevented by WFA. As shown in Figure 1A, the Ang II infusion model was established to continuously release Ang II for a period of 4 weeks.

To analyze the changes in heart functions in response to the continuous infusion of Ang II, we performed transthoracic echocardiography (Echo). The continuous infusion of Ang II (Ang II–vehicle) was associated with a significant increase in LV posterior wall (LVPW) thickness in Ang II-infused/vehicle-treated mice compared to saline-infused mice. Treatment of Ang II-infused mice with WFA resulted in a significant reduction in LVPW thickness compared to Ang II-infused/vehicle-treated mice (Figure 1B). No difference in LVPW was observed in saline-infused mice treated with either vehicle or WFA, suggesting the development of cardiac hypertrophy in mice infused with Ang II and its reversal on treatment with WFA. The measurement of LV fractional shortening (LVFS) in the parasternal long-axis view (LVFSLAX) showed a significant reduction in FS in Ang II-infused/vehicle-treated mice (Figure 1C; $\bar{x}$ = 20%; n = 6) compared to the saline–vehicle group (Figure 1C; $\bar{x}$ = 39.1%; n = 6). Treatment of mice infused with Ang II with WFA significantly increased LVFS to 54.0% when compared to either the Ang II–vehicle mice or saline–vehicle mice, respectively. The same findings were also noted for parasternal short-axis LVFS (LVFSSAX; Figure 1D) measurements. No significant difference was observed between the saline–vehicle mice and the saline–WFA mice.

**Figure 2:**
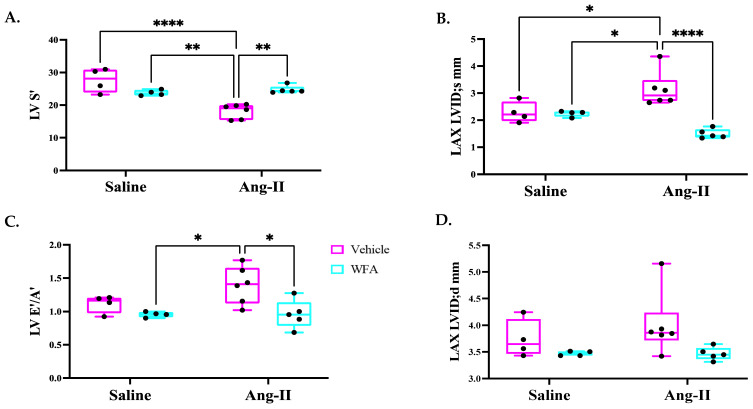
Effect of WFA on diastolic and systolic functions of the left ventricle (LV). The graphical representation of LV mitral annular systolic tissue Doppler S’ (**A**), LV end-systolic internal diameter (LAX LVIDs) in mm (**B**), LV mitral annular tissue Doppler early diastolic velocity E’ and atrial contraction velocity A’ (E’/A’ ratio) (**C**), and LV end-diastolic internal diameter (LAX LVIDd) in mm (**D**). Data are presented as the mean ± SD. * *p* < 0.05; ** *p* < 0.01; **** *p* < 0.0001 indicates a significant difference from the corresponding value of the Ang II-infused group vs. saline-infused group or Ang II–WFA group by two-way ANOVA followed by Tukey’s multiple comparison test. LV = left ventricle; LAX = long axis; ID = internal diameter. Four animals randomly were selected from saline infused groups and 5 to 6 animals randomly were selected from Ang II infused groups for Echo analysis.

LV systolic function was also measured using the LV mitral annulus systolic tissue Doppler velocity S’. The Ang II–vehicle group showed a significant (Figure 2A) decline when compared to the saline–vehicle group. The Ang II–WFA group showed a significant improvement (Figure 2A) when compared to the Ang II–vehicle group, further confirming the beneficial effects of WFA in Ang II-induced LV systolic dysfunctions. Consistent with LVFS, Ang II infusion significantly increased the LV end-systolic internal diameter and WFA treatment was able to restore this to control levels (Figure 2B).

LV diastolic function was assessed using the LV mitral annular tissue Doppler early diastolic velocity E’ and atrial contraction velocity A’ (E’/A’ ratio). The Ang II vehicle group showed a significantly higher E’/A’ ratio, which was reversed by WFA to similar levels observed in saline–vehicle and saline–WFA mice (Figure 2C). However, there were no statistical differences among the four groups in terms of the LV end-diastolic internal diameter (Figure 2D).

**Figure 3:**
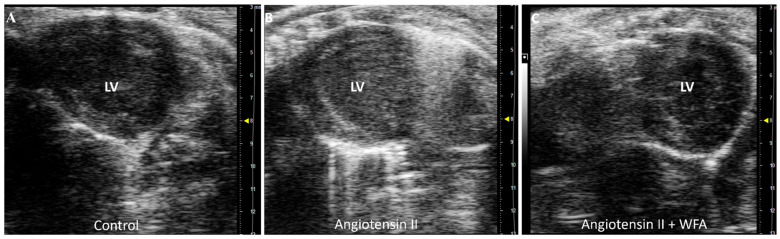
Video 1. Effect of WFA on contractility of LV. WFA significantly increased and restored LV systolic function, as shown in the parasternal short-axis view of transthoracic echocardiography. (**A**) Control mice which showed normal LV systolic function; (**B**) Angiotensin II/vehicle mice which demonstrated reduced LV systolic function; and (**C**) WFA + Ang II mice which showed significantly increased LV systolic function, even higher than the control mice. No difference in LV systolic function between saline-infused and saline–WFA groups. LV = left ventricle. Video S1 included in the Supplementary Materials. Dimension = 1 mm.

The scanning of the LV using echocardiography showed significant damage to LV, associated with the abnormal contractility of the LV in Ang II-infused mice compared to saline-infused mice. Treatment of mice infused with Ang II with WFA completely abrogated the damages and restored LV contractility to or above control levels (Figure 3, Supplementary Materials Video S1). These results suggest the induction of cardiac dysfunction by the continuous infusion of Ang II and its reversal by WFA. No difference in LV contractility was observed between the saline–vehicle and saline–WFA groups.

### 3.2. WFA Reverses the Cardiac Remodeling Induced by Ang II

It remains unknown if all cardiac dysfunctions, particularly those detected by echocardiography, are derived from cardiac structural remodeling, either hypertrophy or fibrosis—both impact cardiac contractility and diastolic capacities, and blood outflow volume. Moreover, Ang II induces physiological changes such as vasoconstriction and cell death, associated with impaired mitochondrial function and structural remodeling (fibrosis and both cardiomyocyte and cardiac hypertrophy) [31]. Therefore, to understand how WFA reserves the cardiac function of mice infused by Ang II, we examined both cardiac hypertrophy and fibrosis.

#### 3.2.1. WFA Reverses the Cardiac Hypertrophy Induced by Ang II

**Figure 4:**
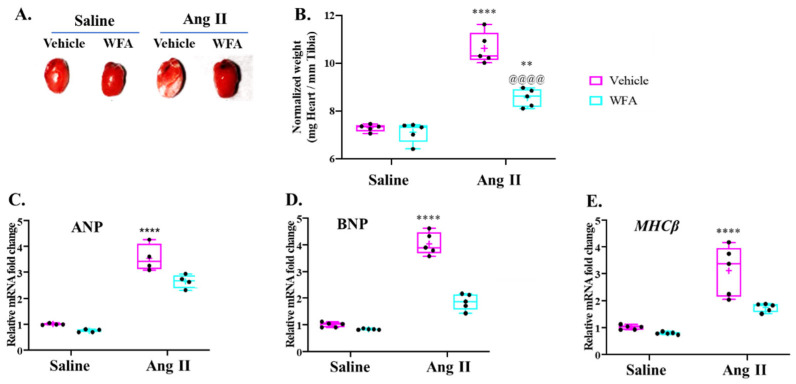
WFA decreases cardiac hypertrophy associated with Ang II-infused mice. (**A**) Hearts were taken out from saline- and Ang II-infused animals, followed by vehicle or WFA treatment, and photographed. (**B**) Normalized heart weights were obtained by dividing the heart weight by the tibial length of each animal in each group. (**C**–**E**) Cardiac mRNA levels of ANP (**C**), BNP (**D**), and MHCβ (**E**) in cardiac tissues. The mRNA levels in each animal were analyzed by qRT-PCR using the specific primers (Table 1). Data are presented as the mean ± SD ((**B**), n = 5; (**C**), n = 4; (**D**,**E**), n = 5). ** *p* < 0.01, and **** *p* < 0.0001 Ang II-infused vs. saline-infused. ^@@@@^ *p* < 0.0001 Ang II-infused vehicle-treated vs. Ang II-infused WFA-treated animals.

In the present study, the effects of Ang II with and without WFA on the heart were examined at the end of the study (week 4). As shown in Figure 4, no change in the normalized heart weights (heart weight/tibial length) of control mice on treatment with WFA compared to mice infused with the vehicle was observed. However, all the animals infused with Ang II showed a significant increase in heart weight and hypertrophy of the heart, reflected by an increase in heart sizes (Figure 4A), as well as normalized heart weight, compared to saline-infused animals. Treatment of Ang II-infused animals with WFA completely attenuated the increase in heart weight (Figure 4A,B). To further elucidate the preventive effect of WFA on the development of cardiac hypertrophy, the expression levels of the ANP, BNP, and MHCβ mRNA as markers of cardiac hypertrophy were tested by qRT-PCR (Figure 4C–E). Treatment with WFA alone in mice infused with saline did not result in any significant changes in these hypertrophic genes; however, the infusion of mice with Ang II showed significant increases in mRNA levels of each of the ANP, BNP, and MHCβ genes. The increased mRNA levels of these genes were significantly reduced by treatment with WFA, particularly the increased mRNA expressions of BNP and MHCβ, which were reduced nearly to control levels (Figure 4C–E).

**Figure 5:**
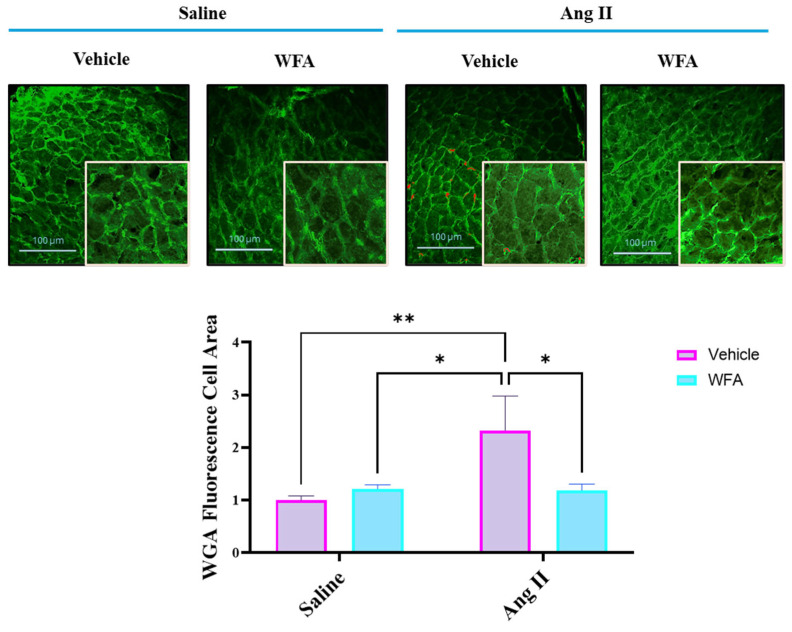
WGA staining and quantification of the relative cardiomyocyte cross-sectional area. Cardiac tissues from two animals from each group were stained with FITC-conjugated WGA. Four to five fields from each tissue were selected, and cross-sectional areas of 15 cells in each field were measured. Data are presented as the mean ± SD. * *p* < 0.05 and ** *p* < 0.01. Scale bar = 100 µM.

WGA staining of the myocytes showed a significant increase in cross-sectional areas of the cardiac muscle cells in Ang II-infused vehicle-treated mice compared to control saline-infused and vehicle- or WFA-treated mice (Figure 5). WFA treatment of mice infused with Ang II significantly reduced the cross-sectional areas. Together, our results clearly demonstrate a significant increase in hypertrophy of the heart by Ang II, which was reversed by WFA.

#### 3.2.2. WFA Reduces the Fibrosis in Cardiac Tissue Induced by Ang II

**Figure 6:**
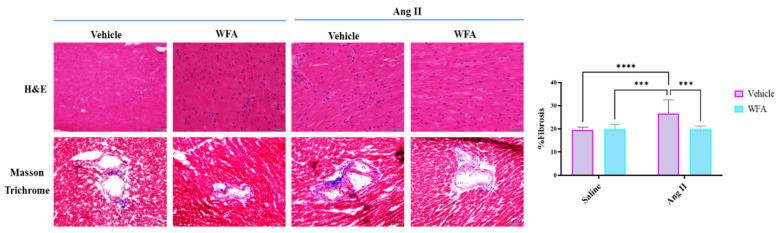
WFA inhibits Ang II-induced cardiac fibrosis. Mice were continuously infused with Ang II or saline as described in Materials and Methods. Cardiac tissues were obtained at the end of the study. Tissues were sectioned and stained with H&E and Mason’s trichrome. Collagen-positive areas (blue color) were identified and quantitated. Data are presented as the mean ± SD (n = 10). *** *p* < 0.001, and **** *p* < 0.0001 Ang II-infused vs. saline-infused. Magnification = 40×.

To decipher the impairment of the cardiac tissue by the Ang II induction and restoration by WFA, we performed a histology of the cardiac tissues using Mason’s trichrome stain to determine perivascular fibrosis. Several studies have pointed out the relationship between perivascular fibrosis and its role in the development of cardiac dysfunction [32,33] and the induction of perivascular fibrosis by elevated Ang II [34]. Consistent with these findings, Ang II infused in mice significantly increased fibrosis in the perivascular regions of the heart compared to control (Figure 6). Treatment with WFA significantly restored these morphological changes (Figure 6). Altogether, our results clearly show that Ang II induces pathological changes to the myocardium, and treatment with WFA ameliorates these morphological changes.

### 3.3. Key Potential Mechanisms Responsible for Ang II-Induced Cardiac Dysfunction and Structural Remodeling

#### 3.3.1. WFA Reverses the Expression of Pro-Inflammatory Cytokines in the Heart

**Figure 7:**
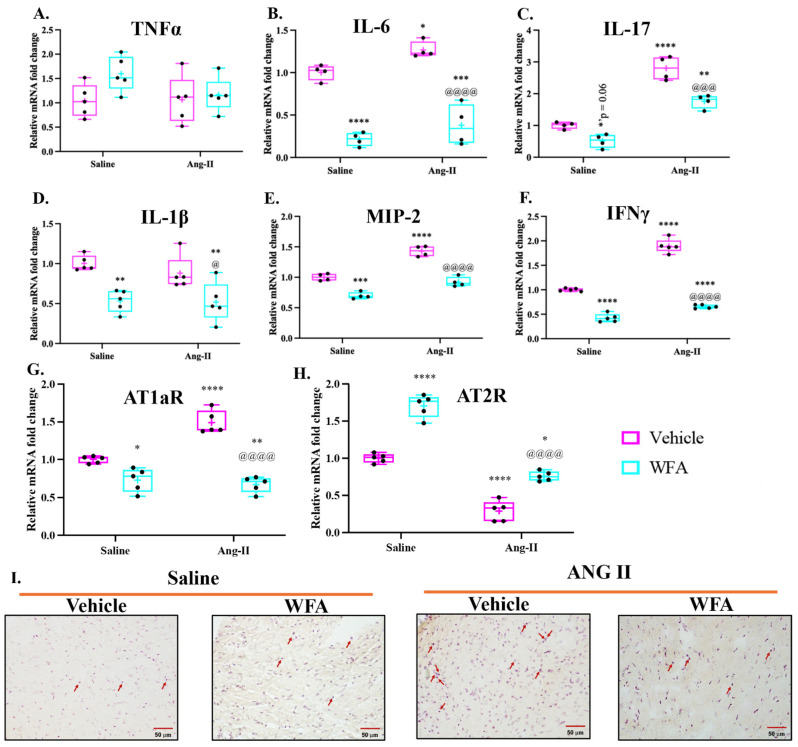
Ang II infusion induces activation of inflammatory cytokine expression in cardiac tissues. Mice were infused with Ang II as described in Materials and Methods. After 4 weeks of infusion, hearts from each group of animals were collected. The expression levels of TNFα (**A**), IL-6 (**B**), IL-17 (**C**), IL-1β (**D**), MIP-2 (**E**), IFN-γ (**F**), AT-1aR (**G**), and AT-2R (**H**) mRNA were quantified by qRT-PCR. Withaferin A impedes the activation of inflammatory cytokines in response to Ang II infusion. Data are presented as the mean ± SD (n = 4–5). * *p* < 0.05, ** *p* < 0.01, *** *p* < 0.001, and **** *p* < 0.0001 Ang II-infused vs. saline-infused. ^@^ *p* < 0.05, ^@@@^ *p* < 0.001, ^@@@@^ *p* < 0.0001 Ang II-infused vehicle-treated vs. Ang II-infused and WFA-treated F4/80 antibody was used to identify the macrophages (**I**). Macrophages in cardiac tissues are indicated by arrows. Scale bar = 50 µm.

The induction of cardiac remodeling by Ang II is a complex mechanism, which is associated with Ang II-increased systemic and cardiac inflammatory cytokines leading to cardiac inflammation in the heart [27,29,35]; therefore, we analyzed the expression of these pro-inflammatory cytokines in the heart in response to Ang II infusion, followed by treatment with WFA. As shown in Figure 7, relative mRNA levels of IL-6 (Figure 7B), IL-17 (Figure 7C), MIP2 (mouse ortholog of human IL-8, Figure 7E), and IFN-γ (Figure 7F) were significantly increased in cardiac tissues of Ang II-infused mice compared to saline-infused mice, and treatment with WFA significantly reduced the expression of these genes to control levels (Figure 7). An increase in TNF and IL-1β were noticed in Ang II-infused animals (Figure 7A,D), respectively, but the increase in levels was not significant compared to saline-infused mice. A significant reduction in the levels of IL-1β was observed upon treatment of Ang-infused animals with WFA. These results suggest that several cardiac inflammatory cytokines that signify the induction of cardiac disorders and morphological changes are involved in mediating the function of Ang II and are targeted by WFA. Ang II infusion also significantly increased AT1a receptor and decreased AT2 receptor mRNA expression levels compared to control mice, while WFA treatment significantly decreased AT1a and increased AT2 receptor mRNA expression levels in both control and Ang II-infused mice (Figure 7G,H).

Our results (Figure 7) clearly indicate the changes in proinflammatory cytokines (TNF-α, IL-6, Il-1β, and others), which are released mainly from M1 macrophages. To confirm the increase in the infiltration of macrophages in cardiac tissues on the continuous infusion of Ang II, we used F4/80-specific antibody to identify the macrophages. As shown in Figure 7I, we observed an increase in the number of macrophages in cardiac tissues from mice infused with Ang II, and such an increase was attenuated on treatment with WFA.

#### 3.3.2. WFA Reverses the Expression of NLRP3 Inflammasomes in Cardiac Muscles

**Figure 8:**
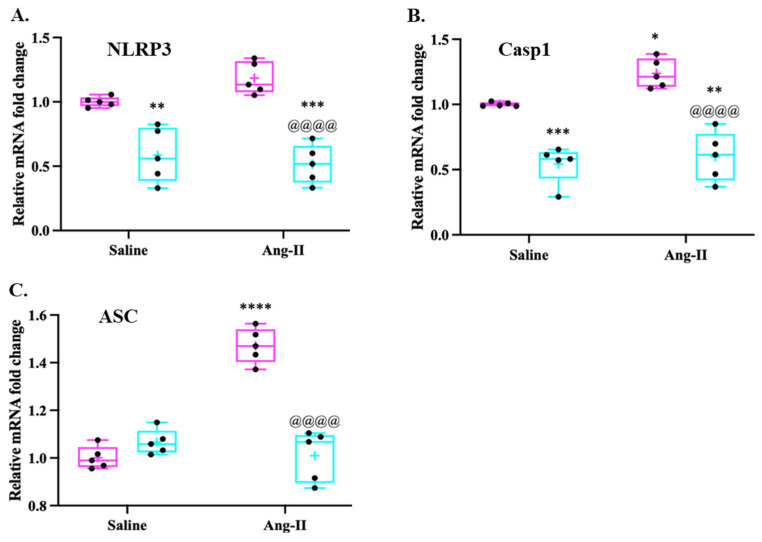
Ang II infusion induces activation of NLRP3 inflammasomes. Mice were infused with Ang II as described in Materials and Methods. After 4 weeks of infusion, hearts from each group of animals were collected. The expression levels of NLRP3 (**A**), Casp1 (**B**), and ASC (**C**) were quantified by qRT-PCR. Data are presented as the mean ± SD (n = 5). * *p* < 0.05, ** *p* < 0.01, *** *p* < 0.001, and **** *p* < 0.0001 Ang II-infused vs. saline-infused animals. ^@@@@^ *p* < 0.0001 Ang II-infused vehicle-treated vs. Ang II-infused WFA-treated animals.

The NOD-like receptor protein 3 (NLRP3) inflammasome comprises the receptor protein NLRP3, apoptosis-related speck-like protein (ASC), and pro-caspase-1 (Casp1) [36]. Blocking the activation of the NLRP3 inflammasome can inhibit Ang II-induced myocardial inflammation, remodeling (hypertrophy and fibrosis), and cardiac dysfunction [35,37]. To determine the induction of NLRP3 inflammasome assembly members in response to Ang II infusion, we evaluated the levels of NLRP3, Casp1, and ASC mRNA using qRT-PCR in cardiac muscles. The continuous infusion of Ang II in mice resulted in an increase in the levels of NLRP3 mRNA (Figure 8A), and a significant increase in the levels of Casp1 mRNA (Figure 8B) and ASC (Figure 8C) compared to saline-infused mice. WFA treatment resulted in a significant reduction in the levels of NLRP3, Casp1, and ASC mRNA levels compared to Ang II–vehicle animals. A significant reduction in the levels of NLRP3 and Casp1 mRNA levels was observed in Ang II on treatment with WFA compared to saline-infused animals when treated with WFA. These results strongly suggest that WFA targets NRP3 pathways that are involved in the induction of myocardial remodeling in response to Ang II.

**Figure 9:**
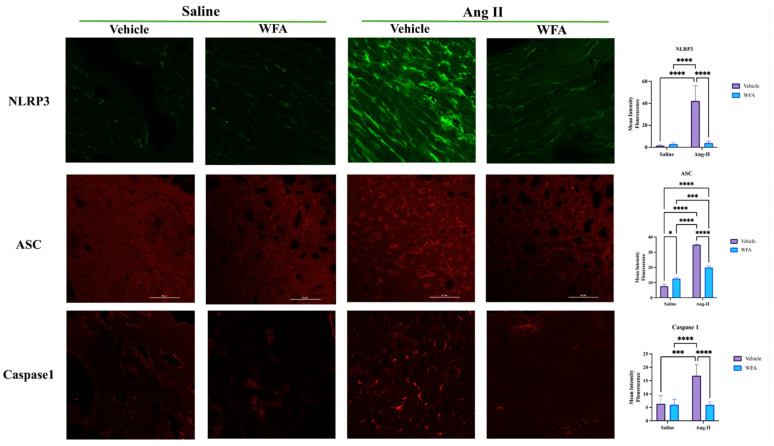
Ang II infusion induces expression of NLRP3, ASC, and Caspase 1 proteins. Mice were infused with Ang II as described in Materials and Methods. After 4 weeks of infusion, hearts from each group of animals were collected. The expression levels of NLRP3, ASC, and Caspase 1 proteins in cardiac tissues were evaluated by immunofluorescence technique. Data are presented as the mean ± SD (n = 5). * *p* < 0.05, *** *p* < 0.001, and **** *p* < 0.0001 Ang II-infused vs. saline-infused groups. Scale Bar = 50 µm.

Nlrp3, caspase 1, and ASC are the key members of the NLRP3 inflammasome complex and exert their function on the protein levels. Therefore, to confirm the expression levels of NLRP3, ASC, and caspase 1 proteins, we performed immunofluorescence analysis of the cardiac tissues collected from all the groups of animals as described in Materials and Methods. The infusion of Ang II resulted in a significant increase in the levels of expression of all three components of NLRP3 inflammasomes (NLRP3, ASC, and caspase 1) in cardiac tissues compared to the infusion of saline. Treatment of animals with WFA significantly suppressed (almost to normal) the levels of each NLRP3, ASC, and caspase 1 protein compared to saline-infused vehicle-treated animals. No difference in expression levels was observed in mice infused with saline followed by treatment with vehicle or WFA (Figure 9).

## 4. Discussion

Our previous studies showed that WFA was able to reverse the deterioration caused by cancer in both the skeletal muscle and cardiac muscle in ovarian-cancer-xenografted mouse models [13,14]. However, considering that the key inflammatory mediator for cardiac muscle wasting in tumor-bearing mice is most likely due to the Ang II that was directly secreted by tumor cells or systemically generated by non-cardiac tissues stimulated by tumors [19,21,24,38]. These studies could not address whether these restorations by WFA were mediated by its anti-tumorigenic properties that might, in turn, reduce the levels of Ang II or WFA’s direct, inherent anti-cachectic properties. To address this important issue, the present study used a cachexic model induced by continuous exposure to Ang II with pharmacologically implanting osmotic pumps in immune-competent C57BL/6 mice without a tumor to directly answer this question. The developed cardiac cachexia caused by Ang II, shown by cardiac remodeling and dysfunctions, was almost completely prevented or reversed by treatment with WFA started in the last 3 weeks of the total 4 weeks of Ang II infusion.

The first report of cardiac changes in cancer-induced cachexia in mice was reported by Tian et al., in 2010 [39], where the heart showed dysfunction on day 14 after tumor inoculation, reflected by reduced fractional shortening in vivo by echocardiography. At necropsy (on day 17), the hearts of the tumor-bearing mice group displayed significant fibrosis, and further analysis by transmission electron microscopy revealed a disrupted myocardial ultrastructure. The gene expression of troponin I, a regulator of cardiac muscle contraction, was reduced along with mRNA, and protein levels of MHCα decreased and MHCβ increased. These changes were recaptured in our ovarian-cancer-bearing mice [14]. Therefore, cardiac dysfunction in mice with tumor-induced cachexia was associated with an altered composition of contractile proteins of cardiac muscle, increased myocardial fibrosis, and a disrupted cardiac muscle structure, which are universal features of cardiac cachexia rather than a type of tumor-dependent phenomenon. In the present study with the continual infusion of Ang II for 4 weeks, the general features of cardiac cachexia were determined to show a significant decrease in cardiac functions (Figure 1, Figure 2 and Figure 3), structural remodeling, including increased MHCβ mRNA expression levels (Figure 4), and myocardial fibrosis (Figure 6), confirming the success of this model and mimicking the cancer-induced cardiac cachexia model.

**Figure 10:**
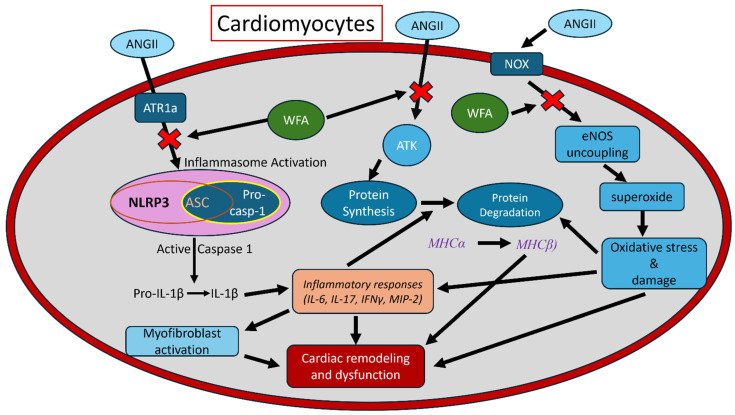
Schematic representation of the proposed Ang II functions in induction of cardiac dysfunction and restoration by WFA. We proposed that high levels of circulatory Ang II can lead to LV systolic and diastolic dysfunction through its specific receptors and regulating several pathways (see details in the discussion). Treatment with WFA ameliorates the effects of Ang II and restores cardiac functions.

As discussed above, it is confirmed that Ang II is an important pathogenic factor to mediate systemic and cardiac cachexia through multiple pathways, as outlined in Figure 10. Ang II, after its binding to type 1 receptor type 1 (AT1R), could stimulate cellular mechanisms including NOXs and uncoupling NOS to generate superoxide and peroxynitrite, which, in turn, cause intracellular oxidative stress and damage to various proteins and enzymes [31,40,41]. Ang II also interacts with the IGF-1 receptor to inhibit Akt/mTOR-mediated protein synthesis and increase protein ubiquitin-mediated protein breakdown to suppress the IGF-1-mediated cachexic switch of MHCα to MHCβ, as a key factor responsible for the loss of cardiomyocyte contractibility [21,38]. Our results confirm that mice infused with Ang II displayed significant functional impairment in both LV systole and diastole measurements. WFA attenuated these conditions in Ang II mice and enhanced contractility compared to saline-infused groups. These effects were associated with enhanced cardiac performance and restoration, suggesting a direct function of WFA in cardiac protection through the upregulation of anti-apoptotic mitochondrial pathways [42]. Cardiac failure is predominantly attributed to the switching of expression of MHC isoforms [39,43] since a small shift in the isoforms MHCα to MHCβ can significantly impair cardiac contractibility. In the adult mouse heart, the primary MHC isoform is MHCα which has a higher ATPase activity than the MHCβ isoform found in the fetal mouse heart. Our results showed a small reduction in the expression levels of MHCα, but a significant increase in the expression levels of MHCβ mRNA in Ang II-induced vehicle-treated mice compared to saline-infused mice (Figure 4). Such an increase in the levels of MHCβ was completely restored to control levels on treatment with WFA.

Morphological changes in the LV were analyzed for perivascular fibrosis using Masson’s trichrome stain. Our results clearly demonstrate that, on a cellular level, Ang-II infused mice showed a significantly higher percentage of perivascular fibrosis. Treatment of animals infused with Ang II with WFA showed a significant reduction in perivascular fibrosis (Figure 6). The inflammatory responses induced by cancers and Ang II are another key player in the development of cardiac cachexia since these inflammatory responses disturb muscle protein synthesis and degradation homeostasis [27,29,35]. Particularly, the cytokine interleukin-6 (IL-6) has been linked to muscle wasting since the injection of IL-6 into rodents induces muscle protein breakdown and IL-6 receptor antibody prevents muscle atrophy in mice that overexpress IL-6 [27,44,45]. Zhang et al. [27] found that circulating levels of IL-6 and serum amyloid-A (SAA) significantly increased in Ang II-infused mice, and IL-6 and SAA synergistically mediated muscle wasting. These investigators reported that Ang II is able to increase SOCS3 to downregulate IRS-1 and insulin/IGF-1 signaling by decreasing Akt phosphorylation, a response that triggers caspase-3 and UPS activation. This finding was further supported by various studies: (1) IL-6-deficient mice are protected from Ang II-induced muscle wasting [24]; (2) Losartan treatment prevented the tumor-induced loss of muscle mass and attenuated the myocardial expression of IL-6 [15]; (3) In heart failure patients, elevated levels of IL-6 were associated with a reduced LV ejection fraction and poor clinical outcomes [46]. Therefore, IL-6 has been proposed as one of the two most essential markers or pathogenic factors for cancer- and cardiac-induced cachexia [7]. In our present study, WFA reduced IL-6 mRNA levels in both saline- and Ang II-infusion groups (Figure 7), suggesting a direct action of WFA on cardiac tissues independent of endogenous conditions. We also demonstrated a remarkable reduction in Ang II-induced inflammatory responses in cardiac tissues with WFA treatment, which resulted in a reduction in cardiac fibrosis (Figure 6 and Figure 7), further confirming a direct function of WFA on cardiac tissues.

Blocking the activation of the NLRP3 inflammasome can inhibit Ang II-induced myocardial inflammation, remodeling (hypertrophy and fibrosis), and cardiac dysfunction [35,37]. In support of the important role of NLRP3, as well as its key components caspase 1 and ASC, Ang II-induced cardiac dysfunction and structural remodeling are associated with a remarkable increase in ASC mRNA and caspase 1 mRNA expression levels (Figure 8 and Figure 9); however, WFA significantly reduced the mRNA and protein expression of caspase 1 in both saline- and Ang II-infusion groups, but only remarkably reduced ASC mRNA expression. Gan et al. [35] reported that Ang II stimulates NLRP3 inflammasome formation, activating caspase-1 from pro-caspase-1. The active caspase-1 activates IL-1β from pro-IL-1β to upregulate inflammatory responses, including IL-6, IL-17, and IFNγ, as well as MIP-2, all of which cause the activation of the myofibroblast and cardiac remodeling, as well as dysfunction [35]. Several recent studies identified the role of Nlrp3-mediated Ang II-induced cardiac remodeling and dysfunction [37,47,48,49]. Nlrp3 gene deletion attenuated mitochondrial abnormalities, cardiac inflammation, oxidative stress, and fibrosis, and, thus, alleviated heart dysfunction and hypertrophy [37].

Finally, oxidative stress’s critical role in the development of skeletal muscle cachexia is well-appreciated [50]. Regarding this aspect, we have previously shown its important role in Ang II-induced cardiac remodeling and dysfunction under different pathogenic conditions with transgenic mice overexpressing the free radical scavenger, metallothionein [31], and remodel via the prevention of early cell death pathways. Combining our previous studies [14,31] with the findings from the present studies, we proposed a hypothesis on how Ang II induces cardiac cachexia and how WFA prevents Ang II-mediated cardiac inflammation, oxidative stress, and structural remodeling (summarized in Figure 10).

## 5. Conclusions

In our studies, we showed that the infusion of Ang II by pharmacologically implanting osmotic pumps in immune-competent C57BL/6 mice (without tumor), followed by treatment with WFA, showed a significant reduction/reversal in Ang II-induced cardiac remodeling and dysfunctions (systolic and diastolic), likely due to the targeting of multi-pathways, demonstrating a direct anticachectic effect of WFA on the heart (Figure 10). In short, we hypothesize that Ang II plays a major role in mediating cardiac muscle wasting through multiple pathways, such as blocking IGF-1-mediated AKT/mTOR pathways, activating NOXs, and uncoupling NOS-mediated oxidative stress, and, consequently, resulting in the systemic inflammation [19,20,21,23,31]. Several reports noticed the activation of Nrf2 by WFA via a couple of pathways, including the PI3K/Akt and/or Keap1 pathway [51,52,53,54]. WFA upregulated the expression of Nrf2-mediated genes encoding for antioxidant enzymes and peptides, including metallothionein, to protect the heart against several chronic pathogeneses [23,51,53,55,56]. However, due to the limited amount of cardiac tissues, we could not explore these known pathways, but these will remain the focus of our extended studies.

Finally, since there is a lack of diagnostic markers and FDA-approved drugs for the treatment of cachexia, there is, therefore, an urgent need to develop an efficacious therapeutic to treat cachexia, especially cardiac cachexia. WFA appears to be a potential therapeutic to target cachexia in patients with cancer or cardiac disorders. Information obtained from our studies will set up a basis to test the efficacy of WFA in treating tumors and cachexia in clinical trials in humans.

## Figures and Tables

**Table 1:** Sequences of primers.

Gene	Forward	Reverse
β-MHC	5′-CTACAGGCCTGGGCTTAC CT-3′	5′-GCCACAAGCAGGAATGAG AA-3′
TNFα	5′-AGCACAGAAAGCATGATC CG-3′	5′-GCCACAAGCAGGAATGAG AA-3′
IL-6	5′-CCTTCTTGGGACTGATGC TGG-3′	5′-GCCTCCGACTTGTGAAGT GGT-3′
IFNγ	5′-GACAATCAGGCCATCAGC AAC-3′	5′-CGGATGAGCTCATTGAAT GCTT-3′
MIP-2	5′-CCACTCTCAAGGGCGGTC AAA-3′	5′-TACGATCCAGGCTTCCCG GGT-3′
ANP	5′-GCTTCGGGGTAGATTGAC-3′	5′-TAGATGAAGGCAGGAAGCCG-3′
BNP	5′-TTTGGGCTGTAACGCACTGA-3′	5′-TTGTGGCAAGGTTTGTGCTCC-3′
IL-17	5′-ATCCCTCAAAGCTCAGCGTGTC-3′	5′-GGGTCTTCATTGCGGTGGAGAG-3′
IL-1β	5′-TCACAGCAGCACATCAACAA-3′	5′-TGTCCTCATCCTGGAAGGTC-3′
NLRP3	5′-AGAAGAGACCACGGCAGAAG-3′	5′-CCTTGGACCAGGTTCAGTGT-3′
Casp1	5′-CACAGCTCTGGAGATGGTGA-3′	5′-GGTCCCACATATTCCCTCCT-3′
Asc	5′-CTGCTCAGAGTACAGCCAGAA-3′	5′-CTGTCCTTCAGTCAGACACTG-3′
AT-1aR	5′-CATTCCTGGATGTGCTG-3′	5′-GAACAAGACGCAGGCTTT-3′
AT-2R	5′-TTATTACCTGCATGAGTGTCGATAGG-3′	5′-AGATGCTTGCCAGGGATTCC-3′
β-Actin	5′-CAGGCATTGCTGACAGGA TG-3′	5′-TGCTGATCCACATCTGCT GG-3′

## Data Availability

Data are contained within the article will be available.

## Supplementary Materials

The following supporting information can be downloaded at: https://www.mdpi.com/article/10.3390/cells13090783/s1, Supplement data. Video S1. Effect of WFA on contractility of LV.

## References

[1] Roeland E.J., Bohlke K., Baracos V.E., Bruera E., Del Fabbro E., Dixon S., Fallon M., Herrstedt J., Lau H., Platek M. (2020). Management of Cancer Cachexia: ASCO Guideline. J. Clin. Oncol..

[2] von Haehling S., Anker S.D. (2010). Cachexia as a major underestimated and unmet medical need: Facts and numbers. J. Cachexia Sarcopenia Muscle.

[3] Callaway C.S., Mouchantat L.M., Bitler B.G., Bonetto A. (2023). Mechanisms of Ovarian Cancer-Associated Cachexia. Endocrinology.

[4] Belloum Y., Rannou-Bekono F., Favier F.B. (2017). Cancer-induced cardiac cachexia: Pathogenesis and impact of physical activity (Review). Oncol. Rep..

[5] Bora V., Patel B. (2022). Cardiac Complications: The Understudied Aspect of Cancer Cachexia. Cardiovasc. Toxicol..

[6] Hweidi I.M., Al-Omari A.K., Rababa M.J., Al-Obeisat S.M., Hayajneh A.A. (2021). Cardiac cachexia among patients with chronic heart failure: A systematic review. Nurs. Forum.

[7] Nogueira-Ferreira R., Sousa-Nunes F., Leite-Moreira A., Moreira-Costa L., Vitorino R., Lara Santos L., Moreira-Goncalves D., Ferreira R. (2022). Cancer- and cardiac-induced cachexia: Same fate through different inflammatory mediators?. Inflamm. Res..

[8] Tichy L., Parry T.L. (2023). The pathophysiology of cancer-mediated cardiac cachexia and novel treatment strategies: A narrative review. Cancer Med..

[9] Okoshi M.P., Capalbo R.V., Romeiro F.G., Okoshi K. (2017). Cardiac Cachexia: Perspectives for Prevention and Treatment. Arq. Bras. Cardiol..

[10] Hahm E.R., Kim S.H., Singh K.B., Singh K., Singh S.V. (2020). A Comprehensive Review and Perspective on Anticancer Mechanisms of Withaferin A in Breast Cancer. Cancer Prev Res.

[11] Yan Z., Guo R., Gan L., Lau W.B., Cao X., Zhao J., Ma X., Christopher T.A., Lopez B.L., Wang Y. (2018). Withaferin A inhibits apoptosis via activated Akt-mediated inhibition of oxidative stress. Life Sci..

[12] Fong M.Y., Jin S., Rane M., Singh R.K., Gupta R., Kakar S.S. (2012). Withaferin A synergizes the therapeutic effect of doxorubicin through ROS-mediated autophagy in ovarian cancer. PLoS ONE.

[13] Straughn A.R., Kelm N.Q., Kakar S.S. (2021). Withaferin A and Ovarian Cancer Antagonistically Regulate Skeletal Muscle Mass. Front. Cell Dev. Biol..

[14] Kelm N.Q., Straughn A.R., Kakar S.S. (2020). Withaferin A attenuates ovarian cancer-induced cardiac cachexia. PLoS ONE.

[15] Rolfe M., Kamel A., Ahmed M.M., Kramer J. (2019). Pharmacological management of cardiac cachexia: A review of potential therapy options. Heart Fail. Rev..

[16] Stevens S.C., Velten M., Youtz D.J., Clark Y., Jing R., Reiser P.J., Bicer S., Devine R.D., McCarthy D.O., Wold L.E. (2015). Losartan treatment attenuates tumor-induced myocardial dysfunction. J. Mol. Cell Cardiol..

[17] Scherrer-Crosbie M. (2015). Losartan: A new treatment for cardiac cachexia?. J. Mol. Cell Cardiol..

[18] Yoshida T., Delafontaine P. (2020). Mechanisms of IGF-1-Mediated Regulation of Skeletal Muscle Hypertrophy and Atrophy. Cells.

[19] Brink M., Wellen J., Delafontaine P. (1996). Angiotensin II causes weight loss and decreases circulating insulin-like growth factor I in rats through a pressor-independent mechanism. J. Clin. Investig..

[20] Kadoguchi T., Shimada K., Koide H., Miyazaki T., Shiozawa T., Takahashi S., Aikawa T., Ouchi S., Kitamura K., Sugita Y. (2018). Possible Role of NADPH Oxidase 4 in Angiotensin II-Induced Muscle Wasting in Mice. Front. Physiol..

[21] Delafontaine P., Akao M. (2006). Angiotensin II as candidate of cardiac cachexia. Curr. Opin. Clin. Nutr. Metab. Care.

[22] Sanders P.M., Russell S.T., Tisdale M.J. (2005). Angiotensin II directly induces muscle protein catabolism through the ubiquitin-proteasome proteolytic pathway and may play a role in cancer cachexia. Br. J. Cancer.

[23] Russell S.T., Eley H., Tisdale M.J. (2007). Role of reactive oxygen species in protein degradation in murine myotubes induced by proteolysis-inducing factor and angiotensin II. Cell Signal..

[24] Yoshida T., Tabony A.M., Galvez S., Mitch W.E., Higashi Y., Sukhanov S., Delafontaine P. (2013). Molecular mechanisms and signaling pathways of angiotensin II-induced muscle wasting: Potential therapeutic targets for cardiac cachexia. Int. J. Biochem. Cell Biol..

[25] Penafuerte C.A., Gagnon B., Sirois J., Murphy J., MacDonald N., Tremblay M.L. (2016). Identification of neutrophil-derived proteases and angiotensin II as biomarkers of cancer cachexia. Br. J. Cancer.

[26] Yamauchi A., Kamiyoshi A., Sakurai T., Miyazaki H., Hirano E., Lim H.S., Kaku T., Shindo T. (2019). Placental extract suppresses cardiac hypertrophy and fibrosis in an angiotensin II-induced cachexia model in mice. Heliyon.

[27] Zhang L., Du J., Hu Z., Han G., Delafontaine P., Garcia G., Mitch W.E. (2009). IL-6 and serum amyloid A synergy mediates angiotensin II-induced muscle wasting. J. Am. Soc. Nephrol..

[28] Song Y.H., Li Y., Du J., Mitch W.E., Rosenthal N., Delafontaine P. (2005). Muscle-specific expression of IGF-1 blocks angiotensin II-induced skeletal muscle wasting. J. Clin. Investig..

[29] Shen C., Zhou J., Wang X., Yu X.Y., Liang C., Liu B., Pan X., Zhao Q., Song J.L., Wang J. (2017). Angiotensin-II-induced Muscle Wasting is Mediated by 25-Hydroxycholesterol via GSK3beta Signaling Pathway. EBioMedicine.

[30] Zhang G., Kang Y., Cathey D., LeBlanc A.J., Cai J., Cai L., Wang S., Huang J., Keller B.B. (2022). Sulforaphane Does Not Protect Right Ventricular Systolic and Diastolic Functions in Nrf2 Knockout Pulmonary Artery Hypertension Mice. Cardiovasc. Drugs Ther..

[31] Zhou G., Li X., Hein D.W., Xiang X., Marshall J.P., Prabhu S.D., Cai L. (2008). Metallothionein suppresses angiotensin II-induced nicotinamide adenine dinucleotide phosphate oxidase activation, nitrosative stress, apoptosis, and pathological remodeling in the diabetic heart. J. Am. Coll. Cardiol..

[32] Zhuang R., Chen J., Cheng H.S., Assa C., Jamaiyar A., Pandey A.K., Pérez-Cremades D., Zhang B., Tzani A., Wara A.K. (2022). Perivascular Fibrosis Is Mediated by a KLF10-IL-9 Signaling Axis in CD4+ T Cells. Circ. Res..

[33] Ytrehus K., Hulot J.S., Perrino C., Schiattarella G.G., Madonna R. (2018). Perivascular fibrosis and the microvasculature of the heart. Still hidden secrets of pathophysiology?. Vascul Pharmacol..

[34] Broekmans K., Giesen J., Menges L., Koesling D., Russwurm M. (2020). Angiotensin II-Induced Cardiovascular Fibrosis Is Attenuated by NO-Sensitive Guanylyl Cyclase1. Cells.

[35] Gan W., Ren J., Li T., Lv S., Li C., Liu Z., Yang M. (2018). The SGK1 inhibitor EMD638683, prevents Angiotensin II-induced cardiac inflammation and fibrosis by blocking NLRP3 inflammasome activation. Biochim. Biophys. Acta Mol. Basis Dis..

[36] Sharma D., Kanneganti T.D. (2016). The cell biology of inflammasomes: Mechanisms of inflammasome activation and regulation. J. Cell Biol..

[37] Chen Y., Zeng M., Zhang Y., Guo H., Ding W., Sun T. (2021). Nlrp3 Deficiency Alleviates Angiotensin II-Induced Cardiomyopathy by Inhibiting Mitochondrial Dysfunction. Oxidative Med. Cell. Longev..

[38] Saha S., Singh P.K., Roy P., Kakar S.S. (2022). Cardiac Cachexia: Unaddressed Aspect in Cancer Patients. Cells.

[39] Tian M., Nishijima Y., Asp M.L., Stout M.B., Reiser P.J., Belury M.A. (2010). Cardiac alterations in cancer-induced cachexia in mice. Int. J. Oncol..

[40] Liu Q., Wang G., Zhou G., Tan Y., Wang X., Wei W., Liu L., Xue W., Feng W., Cai L. (2009). Angiotensin II-induced p53-dependent cardiac apoptotic cell death: Its prevention by metallothionein. Toxicol. Lett..

[41] Tan Y., Li X., Prabhu S.D., Brittian K.R., Chen Q., Yin X., McClain C.J., Zhou Z., Cai L. (2012). Angiotensin II plays a critical role in alcohol-induced cardiac nitrative damage, cell death, remodeling, and cardiomyopathy in a protein kinase C/nicotinamide adenine dinucleotide phosphate oxidase-dependent manner. J. Am. Coll. Cardiol..

[42] Guo R., Gan L., Lau W.B., Yan Z., Xie D., Gao E., Christopher T.A., Lopez B.L., Ma X., Wang Y. (2019). Withaferin A Prevents Myocardial Ischemia/Reperfusion Injury by Upregulating AMP-Activated Protein Kinase-Dependent B-Cell Lymphoma2 Signaling. Circ. J..

[43] Cosper P.F., Leinwand L.A. (2011). Cancer causes cardiac atrophy and autophagy in a sexually dimorphic manner. Cancer Res..

[44] Tsujinaka T., Fujita J., Ebisui C., Yano M., Kominami E., Suzuki K., Tanaka K., Katsume A., Ohsugi Y., Shiozaki H. (1996). Interleukin 6 receptor antibody inhibits muscle atrophy and modulates proteolytic systems in interleukin 6 transgenic mice. J. Clin. Investig..

[45] Goodman M.N. (1994). Interleukin-6 induces skeletal muscle protein breakdown in rats. Proc. Soc. Exp. Biol. Med..

[46] Markousis-Mavrogenis G., Tromp J., Ouwerkerk W., Devalaraja M., Anker S.D., Cleland J.G., Dickstein K., Filippatos G.S., van der Harst P., Lang C.C. (2019). The clinical significance of interleukin-6 in heart failure: Results from the BIOSTAT-CHF study. Eur. J. Heart Fail..

[47] Espitia-Corredor J.A., Boza P., Espinoza-Perez C., Lillo J.M., Rimassa-Tare C., Machuca V., Osorio-Sandoval J.M., Vivar R., Bolivar S., Pardo-Jimenez V. (2022). Angiotensin II Triggers NLRP3 Inflammasome Activation by a Ca(2+) Signaling-Dependent Pathway in Rat Cardiac Fibroblast Ang-II by a Ca(2+)-Dependent Mechanism Triggers NLRP3 Inflammasome in CF. Inflammation.

[48] Liu Y., Bi X., Zhang Y., Wang Y., Ding W. (2020). Mitochondrial dysfunction/NLRP3 inflammasome axis contributes to angiotensin II-induced skeletal muscle wasting via PPAR-gamma. Lab. Invest..

[49] Ren B., Feng J., Yang N., Guo Y., Chen C., Qin Q. (2021). Ginsenoside Rg3 attenuates angiotensin II-induced myocardial hypertrophy through repressing NLRP3 inflammasome and oxidative stress via modulating SIRT1/NF-kappaB pathway. Int. Immunopharmacol..

[50] Sukhanov S., Semprun-Prieto L., Yoshida T., Michael Tabony A., Higashi Y., Galvez S., Delafontaine P. (2011). Angiotensin II, oxidative stress and skeletal muscle wasting. Am. J. Med. Sci..

[51] Heyninck K., Sabbe L., Chirumamilla C.S., Szarc Vel Szic K., Vander Veken P., Lemmens K.J.A., LahtelaKakkonen M., Naulaerts S., Op de Beeck K., Laukens K. (2016). Withaferin A induces heme oxygenase (HO-1) expression in endothelial cells via activation of the Keap1/Nrf2 pathway. Biochem. Pharmacol..

[52] Palliyaguru D.L., Chartoumpekis D.V., Wakabayashi N., Skoko J.J., Yagishita Y., Singh S.V., Kensler T.W. (2016). Withaferin A induces Nrf2-dependent protection against liver injury: Role of Keap1-independent mechanisms. Free Radic. Biol. Med..

[53] Checker R., Bhilwade H.N., Nandha S.R., Patwardhan R.S., Sharma D., Sandur S.K. (2023). Withaferin A, a steroidal lactone, selectively protects normal lymphocytes against ionizing radiation induced apoptosis and genotoxicity via activation of ERK/Nrf-2/HO-1 axis. Toxicol. Appl. Pharmacol..

[54] Zhang Y., Tan Y., Liu S., Yin H., Duan J., Fan L., Zhao X., Jiang B. (2023). Implications of Withaferin A for the metastatic potential and drug resistance in hepatocellular carcinoma cells via Nrf2-mediated EMT and ferroptosis. Toxicol. Mech. Methods.

[55] Zhou S., Yin X., Jin J., Tan Y., Conklin D.J., Xin Y., Zhang Z., Sun W., Cui T., Cai J. (2017). Intermittent hypoxia-induced cardiomyopathy and its prevention by Nrf2 and metallothionein. Free Radic. Biol. Med..

[56] Gu J., Cheng Y., Wu H., Kong L., Wang S., Xu Z., Zhang Z., Tan Y., Keller B.B., Zhou H. (2017). Metallothionein Is Downstream of Nrf2 and Partially Mediates Sulforaphane Prevention of Diabetic Cardiomyopathy. Diabetes.

