# Supplementary material for: Withaferin A as a Potential Therapeutic Target for the Treatment of Angiotensin II-Induced Cardiac Cachexia
Source: Cells. 2024 May 3;13(9):783. doi: 10.3390/cells13090783 (PMC11083229; doi:10.3390/cells13090783)
Supplement: Supplementary file 1 [file cells-13-00783-s001.zip › cells-2841439-supplementary.pptx]

## Slide 1
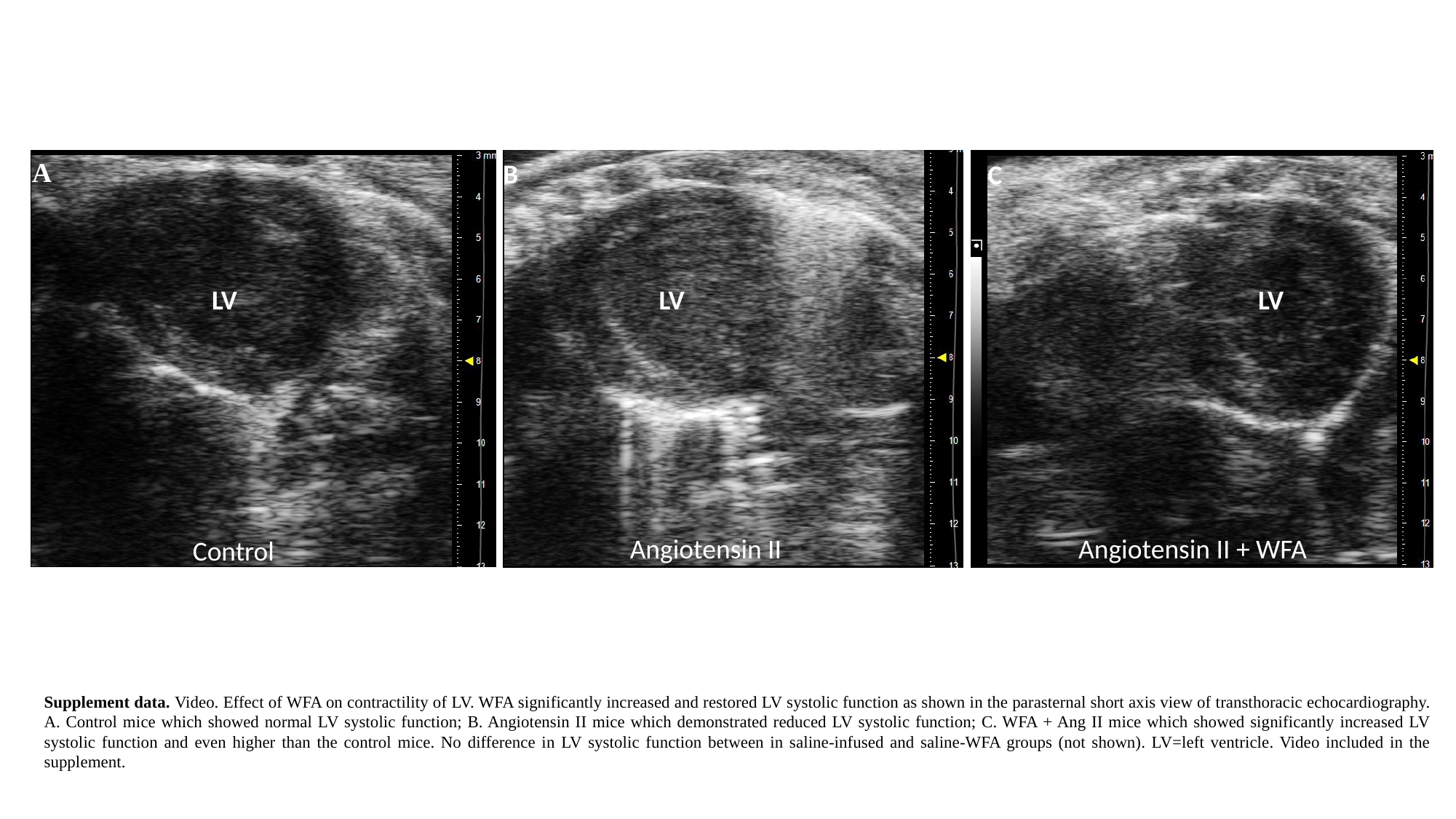

A
B
C
LV
LV
LV
LV
Angiotensin II
Angiotensin II + WFA
Control
Supplement data. Video. Effect of WFA on contractility of LV. WFA significantly increased and restored LV systolic function as shown in the parasternal short axis view of transthoracic echocardiography. A. Control mice which showed normal LV systolic function; B. Angiotensin II mice which demonstrated reduced LV systolic function; C. WFA + Ang II mice which showed significantly increased LV systolic function and even higher than the control mice. No difference in LV systolic function between in saline-infused and saline-WFA groups (not shown). LV=left ventricle. Video included in the supplement.
